# Current Insights into the Modulation of Oral Bacterial Degradation of Dental Polymeric Restorative Materials

**DOI:** 10.3390/ma10050507

**Published:** 2017-05-06

**Authors:** Ning Zhang, Yansong Ma, Michael D. Weir, Hockin H. K. Xu, Yuxing Bai, Mary Anne S. Melo

**Affiliations:** 1 Department of Orthodontics, School of Stomatology, Capital Medical University, Beijing 100050, China; dentistzhang112@163.com (N.Z.); mayansong0911@126.com (Y.M.); 2 Biomaterials & Tissue Engineering Division, Department of Endodontics, Prosthodontics and Operative Dentistry, University of Maryland School of Dentistry, Baltimore, MD 21201, USA; MWeir@umaryland.edu (M.D.W.); hxu@umaryland.edu (H.H.K.X.); 3 Center for Stem Cell Biology & Regenerative Medicine, University of Maryland School of Medicine, Baltimore, MD 21201, USA; 4 Marlene and Stewart Greenebaum Cancer Center, University of Maryland School of Medicine, Baltimore, MD 21201, USA; 5 Department of Mechanical Engineering, University of Maryland, Baltimore, Baltimore, MD 21250, USA; 6 Operative Dentistry Division, Department of General Dentistry, University of Maryland School of Dentistry, Baltimore, MD 21201, USA

**Keywords:** biofilm, dental materials, degradation, dental caries

## Abstract

Dental polymeric composites have become the first choice for cavity restorations due to their esthetics and capacity to be bonded to the tooth. However, the oral cavity is considered to be harsh environment for a polymeric material. Oral biofilms can degrade the polymeric components, thus compromising the marginal integrity and leading to the recurrence of caries. Recurrent caries around restorations has been reported as the main reason for restoration failure. The degradation of materials greatly compromises the clinical longevity. This review focuses on the degradation process of resin composites by oral biofilms, the mechanisms of degradation and its consequences. In addition, potential future developments in the area of resin-based dental biomaterials with an emphasis on anti-biofilm strategies are also reviewed.

## 1. Introduction

**Figure 1:**
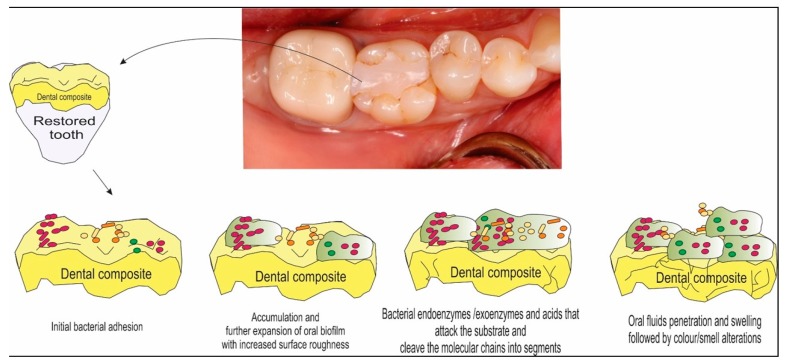
Schematic illustration of a composite surface representing the clinical view of a tooth restored with dental restorative esthetic materials. This sequence of drawings shows the complex interactions between the material’s surface and the biofilm formation over time.

Dental restorative materials, including metals, polymers, amalgam alloys, ceramics and resin composites, are used to reconstruct the tooth when its structure is compromised by trauma or dental caries [1]. Currently, the most used materials to restore a cavity are resin composites due to their direct-filling capability, esthetics, low toxicity and improved performance [2,3]. However, despite their superiority, resin composites tend to accumulate more biofilm than other restorative materials (Figure 1) [4,5]. This fact has been strongly correlated to the formation of recurrent caries, which has been pointed out as the predominant reason for the failure of composite restorations [2,3,4,5]. The occurrence of recurrent caries is prevalent worldwide with significant health and financial burdens [6].

The oral cavity, which includes bacteria, high forces, changing pH and a warm, fluid environment, is considered to be a harsh environment for dental restorative materials [7,8]. The polymeric materials used for dental restorations can be degraded by various environmental factors such as thermal, oxidative, hydrolytic and mechanical. As a major pathological factor, oral bacteria, especially acidogenic bacteria, can form biofilms and produce acids that can dissolve tooth structure and degrade dental restorative materials, such as resin composites and bonding agents [9]. Of particular concern is the fact that the degradation process of these materials can promote the occurrence of recurrent caries [10]. Therefore, the ability of restorative materials to withstand biofilms is an important requirement for their clinical performance. Information on degradability can provide fundamental understanding to facilitate the design and lifetime analysis of restorative materials.

The scope of this review is confined to the biofilm degradation of polymeric materials that are used for direct esthetic tooth restorations, with the focus on the main issues, such as the consequences of oral biofilm degradation on the performance of these materials, and on future advances in dental biomaterials.

## 2. Concept for Restorative Polymeric Direct Materials

Resin composites consist of a light-polymerizable matrix based on methacrylate monomers containing inorganic fillers, such as silica glass (SiO_2_), alumina glass (Al_2_O_3_) and combinations of glass and sodium fluoride [7]. 2,2-*bis*(4(2-hydroxy-3-methacryloxypropoxy)-phenyl) propane, also known as bisphenol A glycidyl methacrylate (BisGMA), and diluent monomer triethylene glycol methacrylate (TEGDMA) are among the most often used monomers in current resin composites [7]. BisGMA monomer contains a core based on hydrophobic aromatic rings and the two pendant hydroxyl groups, which are slightly hydrophilic and responsible for the high water sorption. It presents strong hydrogen bonding that promotes extremely high viscosity with low chain mobility and less deformation upon mechanical loading relative to linear non-aromatic monomers [10]. For a direct clinical application, enhanced polymeric matrix mobility for material handling is required. The high viscosity of BisGMA necessitates blending with diluent monomers to achieve high filler loading for better physical properties of the composite [11].TEGDMA is a low molecular weight *di*-vinyl monomer that enhances the efficiency of polymerization by reducing the overall viscosity [12]. However, TEGDMA leads to higher water uptake due to the triethylene oxide spacers and higher polymerization shrinkage. The stress generated by volumetric shrinkage at the tooth-restoration interface results in the potential for marginal gaps, which adversely influence the longevity of a resin composite restoration [13]. Most of the currently available resin-based formulations of direct restorative materials are based on Bis-GMA/TEGDMA and do not produce satisfactory durability and esthetics over time. Bis-GMA/TEGDMA resin-based materials contain undesirable ester groups that can eventually be breakdown by acidic, basic or enzymatic-induced hydrolysis [14,15,16].

## 3. Oral Biofilm Degradation of Polymeric Restorative Dental Materials

Oral biofilms are aggregates of microorganisms, which are formed due to the attachment of cells to each other and/or to a host surface in an aqueous environment [14]. The factors that influence the biofilm formation are humidity, temperature, pH of the environment or medium, atmospheric conditions and nutrition sources. Biofilm formation starts with the deposition of microorganisms on the surface of the material, followed by growth and spreading of the colonies (Figure 1). The microbial cells are encased in an adhesive matrix produced by the microorganisms of the biofilm, called extracellular polymer substance (EPS), which contains proteins, nucleic acids, lipids and polysaccharides. EPS influences the adhesion to the surface and plays an important role in the protection of the biofilm from the outer environment. The process of establishment of a complex community of microorganisms on surface attachment as a biofilm is known as biofouling or microfouling [17].

The five major damaging mechanisms (both direct and indirect) through which the structure and function of synthetic polymeric materials can be damaged by biofilms were fundamentally summarized by Flemming [17]. These include: (1) coating the surface, masking surface properties and contaminating adjacent media, such as water, (2) increasing the leaching additives and monomers out of the polymer matrix, (3) attack by enzymes or radicals of biological origin to polymer and additives, leading to both embrittlement and loss of mechanical stability, (4) accumulating water and penetrating the polymer matrix with microbial filaments, causing swelling and increased conductivity, and (5) excretion of lipophilic microbial pigments that lead to unwanted colors in the polymer. Due to the complexity of oral biofilm, the resin composites are exposed to most of the discussed degradation mechanisms simultaneously in vivo.

**Figure 2:**
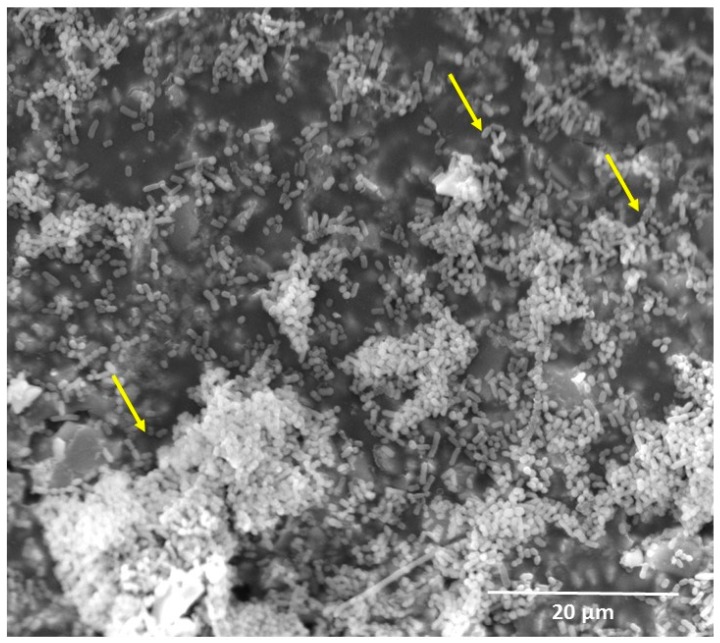
Scanning electron microscope (SEM) image of resin composite covered by oral biofilm (5000×). Observe the abundant bacterial colonies with many streptococcal chains (narrows). *S. mutans*, which composes a significant proportion of the oral streptococci in caries lesions, has been identified as the major etiological agent of human dental caries.

Oral biofilms are highly adaptive to the environment and excrete both endoenzymes and exoenzymes that may accelerate resin composites’ degradation. Enzymatic degradation is the main biodegradation process for several medical polymers [18]. During the degradation, enzymes from microorganisms break down complex polymers yielding short chains or smaller molecules, e.g., oligomers, dimers and monomers, that are small enough (water-soluble) to pass the semi-permeable outer bacterial membranes and then to be utilized as carbon and energy sources [19]. Oral biofilms form not only on dental tissues as the major cause of caries and periodontal diseases [20], but also on restorative material surfaces. Resin composites tend to accumulate more biofilm, compared with other restorations (Figure 2) [4,5]. Several studies have investigated the degradation of resin composites caused by oral biofilms [4,8,21]. Biofilm formation on resin composite not only degrades the material and roughens its surface [4,8], but also causes colonizing bacteria to invade the interface between the restoration and the tooth, leading to recurrent caries [22,23].

Surface roughness is a key property for caries management and is directly related to the plaque accumulation on resin composites, which may influence their longevity. Biofilm acids can cause the surface swelling of resin composites [24], increasing the surface roughness, which in turn, encourages more bacteria to attach to and colonize the resin composite.

To place composite restorations into tooth cavity preparations, it is necessary to use dental primer and adhesive as bonding agents to make the composite adhere to the tooth structure [1,2,3]. The composite and adhesive interfaces are exposed to the oral environment immediately after being light-cured. A perfect seal at the tooth-restoration interfaces is an important goal, but it is often difficult to achieve. Microgaps were often observed at the tooth-restoration margins [22,23]. These microgaps could further deteriorate due to polymerization shrinkage stresses, cyclic fatigue and wear actions [22,23]. These microgaps could accumulate oral biofilms, with acid production leading to recurrent caries.

In addition, the degree of conversion in polymeric matrix greatly affects the physical properties of the restorative materials and may influence the process of degradation [25]. Conventionally, the extent of polymerization is quantified by comparing the amount of remaining double bonds in the polymer structure to the initial amount. This ratio, expressed in percentage (%), is termed the degree of conversion (DC) [25]. The current DC for resin composite ranges around 65%. Thus, monomer conversion is not complete, and at the end of the reaction, part of the monomers remains as unreacted monomers trapped in the polymeric matrix [26]. Approximately 5%–10% of unpolymerized monomer can be extracted in water [27]. It has been suggested that, especially, the release of unreacted monomers from resin composites may enhance the growth of cariogenic bacteria like *S. mutans* and *Lactobacilli*, serving as a source of carbon [27]. The release of these monomers can also enhance the glucosyltransferase activity in *Streptococcus sobrinus* [27].

## 4. Emerging Approaches to Reduce Oral Biofilm Degradation

Biofilm formation over the resin composite has emerged as a major challenge for practitioners nowadays. The anti-biofilm strategies via dental materials to prevent biofilm formation have raised a promising application in combination with traditional methods of biofilm control for the elimination or reduction of problematic oral health condition promoted by the development of recurrent caries around restorations. To overcome these problems, efforts have been devoted to developing a new generation of bioactive dental materials. Development of bioactive resin composites with antibacterial capacity includes two approaches: one is the addition of soluble components that could release bactericidal agent in the oral cavity, and the other one is the incorporation of non-releasing antibacterial components in the material matrix [28,29].

### 4.1. Resin Composite Containing Releasing Antibacterial Agents

Antibacterial agents used in resins include releasing agents and non-releasing agents. Chlorhexidine (CHX), silver (Ag) and fluoride (F) were added into resin or bonding agents as releasing agents [28]. A method was developed to encapsulate and release CHX from composite using mesoporous silica nanoparticles (MSNs) [30]. Another study reported sol-gel bioglass containing silver (Ag-BG), showing antibacterial activity against *Escherichia coli* and *Streptococcus mutans* (*S. mutans*) [31].

CHX is an important antibacterial agent against a wide range of microorganisms [32]. The antibacterial activity of CHX and its uptake by bacteria were dependent on chlorhexidine concentration [32]. Low concentrations of CHX affect membrane integrity, while high concentrations cause cytoplasmic leakage [33]. When contacting CHX, the outer cell membrane of bacteria is damaged rapidly, but this is insufficient to induce cytoplasmic leakage [33]. If there are high concentrations of CHX, then the CHX traverses the outer membrane presumably by passive diffusion and subsequently attacks the bacterial cytoplasmic or inner cell membrane, which leads to the leakage of cytoplasm [32]. In addition, in the oral cavity, the adsorption of salivary proteins on tooth surfaces could produce the acquired pellicle, which is a prerequisite for bacterial attachment and biofilm formation [34]. CHX can combine with saliva glycoprotein, thus reducing protein attachment on tooth surfaces, thereby interfering with the formation of biofilm [32]. Moreover, CHX can also combine with a bacterial extracellular polysaccharide, which makes it difficult for the bacteria to adhere to the acquired pellicle, therefore reducing biofilm formation and preventing dental caries [32].

**Figure 3:**
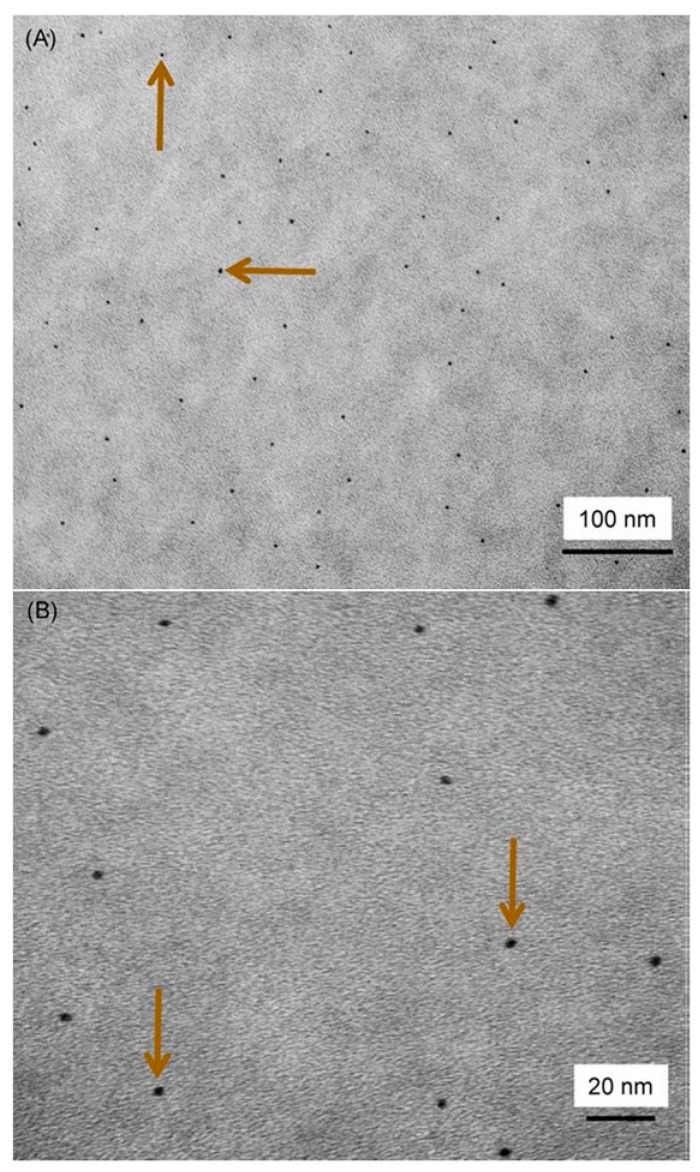
Representative transmission electron microscopy (TEM) micrographs representing the size and dispersion of silver nanoparticles (NAg) in a resin matrix: (**A**) lower and (**B**) higher magnifications. The NAg were formed in the resin by simultaneous reduction of the silver salt and photopolymerization of the dimethacrylates. Arrows indicate the silver nanoparticles, which were well dispersed in the resin with minimal appearance of nanoparticle aggregates. Adapted with permission from [36], copyright SAGE publications, 2012.

Previous studies showed that Ag ions have long-term antibacterial effects and good biocompatibility, low toxicity to human cells and cause less bacterial resistance than antibiotics [35,36]. Regarding the antibacterial mechanism of Ag ions, it was suggested that the Ag ions could inactivate the vital enzymes of bacteria to cause the bacterial DNA to lose its replication ability, which leads to cell death [37]. Nanoparticles of silver (NAg) were shown to have potent antibacterial effects due to their small particle size and high surface area [35,36]. The small particle size and large surface area of NAg could enable them to release more Ag ions at a low filler level, thus decreasing the Ag particle concentration necessary for efficacy (Figure 3) [36]. This is beneficial for dental applications because low Ag filler levels in the material would not compromise the material color and mechanical properties [36]. NAg were recently incorporated into dental resins, which greatly reduced biofilm growth, without affecting the bond strength and material color [35,36].

Although antibacterial agents, such as Ag-, CHX- and fluoride-endowed materials, have antibacterial effects, the release of those agents would lead to weaker mechanical properties and rougher surfaces over time. In addition, the release could have deleterious effects on the environment because of possible toxicity. Furthermore, the release from a resin could adversely influence the mechanical properties due to the voids left.

### 4.2. Resin Composite Containing Non-Releasing Antibacterial Agents

In contrast to soluble antimicrobial agents, non-releasing antimicrobial agents do not leach out from the material, but act as surface contact inhibitors after curing of the composite, being bactericide-immobilized agents with a long-term antibacterial effect [38,39]. Quaternary ammonium salts (QAS) were incorporated into dental resins as non-releasing antimicrobial agents [38,39]. 12-methacryloyloxydodecyl-pyridinium bromide (MDPB) was co-polymerized and covalently bonded in resin to provide durable contact inhibition against bacteria [39]. Other researchers reported quaternary ammonium polyethyleneimine (PEI) nanoparticles in composites [38], methacryloxylethyl cetyl dimethyl ammonium chloride (DMAE-CB)-containing resin composites [40] and, antibacterial nanocomposite using a quaternary ammonium dimethacrylate [41].

**Figure 4:**
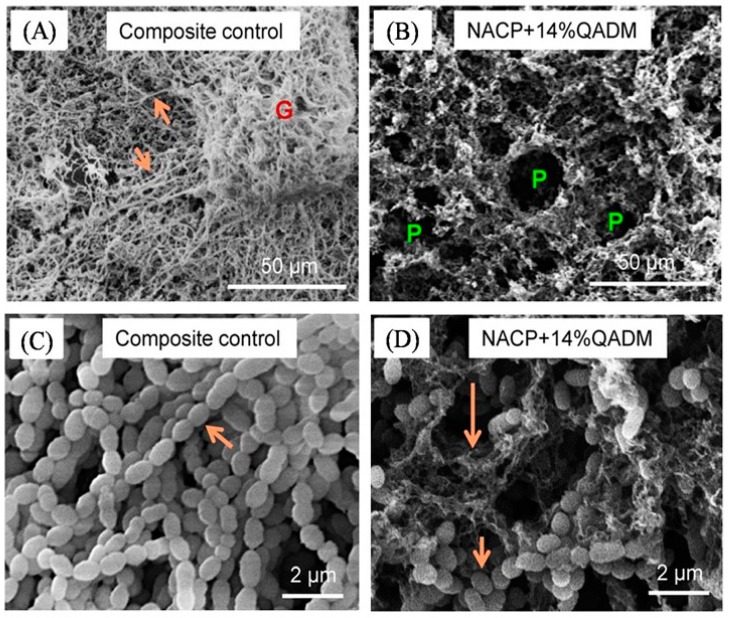
SEM micrographs of plaque microcosm biofilms: (**A**,**B**) lower magnification and (**C**,**D**) higher magnification. In (**A**), the composite control was covered with dense biofilms consisting of numerous long strings (arrows). In (**B**), Quaternary ammonium dimetacrylate associated to nanoparticles of amorphous calcium phosphate (NACP-QADM) nanocomposites had thinner biofilms with numerous pores “P”, without long strings. In (**C**), the long strings were made of bacterial cells connected with each other, and the cells had a normal, healthy short-rod morphology. However, as shown in (**D**), many cells on NACP-QADM nanocomposites had dissolved into pieces, while other cells still had a normal short-rod shape (long arrows indicate cell disintegration, and short arrows indicate normal healthy cells). Adapted with permission from [29], copyright Elsevier 2013.

The antimicrobial mechanism of quaternary ammonium salts (QAS) is via their binding to cell membranes predominantly at the target of the cytoplasmic (inner) membrane in bacteria, which causes cytoplasmic leakage [38,39]. When the negatively-charged bacterial cell contacts the positively-charged (N^+^) sites of QAM resins, the electric balance of the cell membrane could be disturbed, and the bacterium could explode under its own osmotic pressure (Figure 4) [38,39]. Recently, a series of QAMs with alkyl chain length (CL) of 3, 6, 12, 16 and 18 were synthesized [42]. For short-chained QAMs, the antimicrobial activity counts simply on an attraction between the positively-charged ammonium group and the negatively-charged bacterial membrane, having an adverse effect on the balance of essential ions (i.e., K^+^, Na^+^, Ca^2+^ and Mg^2+^), protein activity and bacterial DNA [43]. Increasing CL reduced the metabolic activity and acid of saliva-derived microcosm biofilms. Dimethylaminohexadecyl methacrylate (DMAHDM) with CL 16 showed the strongest antibacterial potency [44]. This was because long-chained quaternary ammonium compounds had double-killing effects: (1) the positive charges; (2) the additional antimicrobial activity by the long alkyl chain inserting into bacterial membranes, resulting in the disruption of bacterial cells [42].

### 4.3. Resin Composite Containing Protein-Repellent Monomers

In the oral environment with salivary flow, a clean dental resin is quickly coated with a salivary pellicle that comprises a layer of selectively adsorbed salivary proteins. It is through this protein layer that oral bacteria attach to the resin and to tooth surfaces [45]. The adherence of early colonizers, for example, *S. mutans*, to the salivary pellicle is an initial step in biofilm formation. Biofilm formation is the source of infection and a prerequisite for the occurrence of dental caries [24,34]. Therefore, it would be highly desirable to develop a resin composite that can repel proteins, to inhibit protein adsorption and hence bacterial adhesion on the surface.

**Figure 5:**
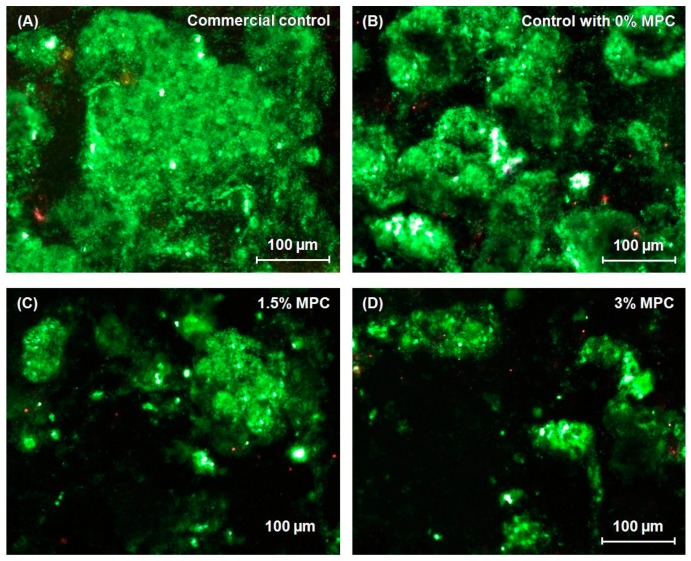
Representative live/dead staining images of biofilms adherent on composite disks cultured for two days: (**A**) commercial control composite; (**B**) control composite with 0% 2-methacryloyloxyethyl phosphorylcholine (MPC); (**C**) control composite with 1.5% MPC and (**D**) control composite with 3% MPC. The live bacteria were stained green, and the dead bacteria were stained red. When live and dead bacteria were in close proximity or on the top of each other, the staining had yellow or orange colors. The composite disks had primarily live bacteria, with few dead bacteria. The commercial control composite (A) and the control composite with 0% MPC (B) had noticeably more bacteria coverage than composites containing MPC. There was less biofilm coverage on control composite disks containing MPC (C,D). The control composite with 3% MPC (D) had the least biofilm coverage. Adapted with permission from [52], copyright Nature Publishing Group 2013.

Overall, there are two kinds of protein-repellent agent. One group is poly(ethylene glycol) (PEG), and the other group is zwitterionic polymers, such as poly(sulfobetaine methacrylate) (pSBMA) and 2-methacryloyloxyethyl phosphorylcholine (MPC) [46,47]. It has been indicated that hydrophilic material surfaces are usually more resistant to protein attachment than hydrophobic surfaces [48]. MPC is a methacrylate with a phospholipid polar group in the side chain [46]. Phospholipids are a major component of all cell membranes, and they can form lipid bilayers [49]. The structure of the phospholipid molecule generally consists of a hydrophilic head (attracted to water) and hydrophobic tails (repelled by water) [49]. When placed in water, phospholipids will orient themselves into a bilayer in which the non-polar tail region faces the inner area of the bilayer. The polar head region faces outward and interacts with the water [46,49]. Hence, the MPC polymers are highly hydrophilic. In the hydrated MPC polymer, there is an abundance of free water, but not bonded water. The presence of bonded water would cause protein adsorption [46,47]. On the other hand, a large amount of free water around the phosphorylcholine group is considered to detach proteins effectively, thereby repelling protein adsorption [46,47]. Various medical devices using MPC have already been developed and clinically used with the approval of the United States Food and Drug Administration (FDA) [50]. A previous study evaluated the durability and antibacterial action of MPC grafting on an acrylic resin-based denture material [51]. The results demonstrated that graft polymerization of MPC on denture surfaces contributed to the durability of the coating and prevented microbial retention. More recently, MPC was successfully incorporated into resin composite, and the novel MPC-based resin composite greatly reduced protein adsorption and bacterial adhesion without compromising mechanical properties (Figure 5) [52].

One limitation of QAM-containing resins is that the salivary protein coating on the resin surface would reduce the efficacy of the “contact-killing” of QAM by reducing the contact of bacteria with the resin [39,53]. It was also reported that the adsorption of salivary proteins on NAg-containing material could decrease its antibacterial activity [53]. This may be due to the antibacterial mode of NAg-containing material, which involves Ag ion release. Salivary proteins could capture the positively-charged Ag ions and work as a barrier to hinder Ag ions’ release [53]. Hence, it would be beneficial to render the resin protein-repellent. It would enhance the antibacterial effectiveness of bioactive dental resins and therefore provide a synergistic effect on biofilm reduction. Indeed, this synergistic effect was confirmed in recent studies [54,55].

### 4.4. Other Approaches to Mitigation of Oral Biofilm Degradation

Besides antibacterial agents incorporated in dental materials, surface modifications are also reported [56]. In these studies, biosurfactants produce by bacteria strains and bacteriophages as a coating to inhibit the biofilm formation on the surfaces [57,58] give the main frame of the research. The efficiency of these materials against biofilm formation was confirmed; in some cases, a 90% growth reduction was observed [57,58]. The mechanism that is responsible for the inhibition of the biofilm is based on the enzymes that are produced by the bacteriophages [56]. These enzymes are capable of destroying the EPS of biofilms [56]. In contrast, biosurfactants are not capable of destroying planktonic cells; they inhibit only the growth of certain strains [58]. Moreover, bacteriophages are only efficient against specific strains, and therefore, the solution might need to have a combination of different biosurfactants and/or bacteriophages [58].

Recently, some studies are focused on improving filler morphology of resin composite by using novel nanoporous alumina filled with silver nanoparticles [59,60]. The alumina microparticles with interconnected nanopores allow mechanical interlocking between fillers and matrix without the need for chemical bonding. This coupling-agent-free dental restorative composite based on nanoporous alumina fillers is promising for being made bioactive after pore filling with different antibacterial agents [59,60].

## 5. Conclusions

Dental restorative esthetic materials used for restorations are subjected to the aggressive attack of oral bacteria. Biodegradation of dental restorative esthetic materials has received a great deal of attention due to its relevance for the development and progression of recurrent caries around the dental restorations. Assessment of biodegradability and strategies to combat it are a key consideration in the development of new restorative dental materials. Research geared towards incorporation of antibacterial agents with different killing approaches with the suitable mechanical properties required for dental restorative esthetic materials seems to provide some future directions for this remaining challenge of restorative dentistry.

## Disclaimer

Official contribution of the National Institute of Standards and Technology (NIST); not subject to copyright in the United States. Certain commercial materials and equipment are identified in this article to specify the experimental procedure. This does not imply recommendation or endorsement by NIST or that the material or equipment identified is necessarily the best available for the purpose.

## References

[1] Ferracane J.L. (2011). Resin composite—State of the art. Dent. Mater..

[2] Drummond J.L. (2008). Degradation, fatigue, and failure of resin dental composite materials. J. Dent. Res..

[3] Bayne S.C. (2012). Correlation of clinical performance with ‘in vitro tests’ of restorative dental materials that use polymer-based matrices. Dent. Mater..

[4] Beyth N., Bahir R., Matalon S., Domb A.J., Weiss E.I. (2008). Streptococcus mutans biofilm changes surface-topography of resin composites. Dent. Mater..

[5] Beyth N., Domb A.J., Weiss E.I. (2007). An in vitro quantitative antibacterial analysis of amalgam and composite resins. J. Dent..

[6] Kassebaum N.J., Bernabé E., Dahiya M., Bhandari B., Murray C.J., Marcenes W. (2015). Global burden of untreated caries: A systematic review and meta regression. J. Dent. Res..

[7] Cramer N.B., Stansbury J.W., Bowman C.N. (2011). Recent advances and developments in composite dental restorative materials. J. Dent. Res..

[8] Busscher H.J., Rinastiti M., Siswomihardjo W., Mei H.C.V.D. (2010). Biofilm formation on dental restorative and implant materials. J. Dent. Res..

[9] Bourbia M., Ma D., Cvitkovitch D.G., Santerre J.P., Finer Y. (2013). Cariogenic bacteria degrade dental resin composites and adhesives. J. Dent. Res..

[10] Delaviz Y., Finer Y., Santerre J.P. (2014). Biodegradation of resin composites and adhesives by oral bacteria and saliva: A rationale for new material designs that consider the clinical environment and treatment challenges. Dent. Mater..

[11] Gajewski V.E., Pfeifer C.S., Fróes-Salgado N.R., Boaro L.C., Braga R.R. (2012). Monomers used in resin composites: Degree of conversion, mechanical properties and water sorption/solubility. Braz. Dent. J..

[12] Goncalves F., Kawano Y.C., Stansbury J.W., Braga R.R. (2009). Influence of BisGMA, TEGDMA, and BisEMA contents on viscosity, conversion, and flexural strength of experimental resins and composites. Eur. J. Oral Sci..

[13] Nagem F.H., Nagem H.D., Francisconi P.A., Franco E.B., Mondelli R.F., Coutinho K.Q. (2007). Volumetric polymerization shrinkage of contemporary composite resins. J. Appl. Oral. Sci..

[14] Spencer P., Ye Q., Park J., Topp E.M., Misra A., Marangos O., Wang Y., Bohaty B.S., Singh V., Sene F. (2010). Adhesive/dentin interface: the weak link in the composite restoration. Ann. Biomed. Eng..

[15] Gonzalez-Bonet A., Kaufman G., Yang Y., Wong C., Jackson A., Huyang G., Bowen R., Sun J. (2015). Preparation of Dental Resins Resistant to Enzymatic and Hydrolytic Degradation in Oral Environments. Biomacromolecules.

[16] Daghighi S., Sjollema J., Hc V.D.M., Busscher H.J., Rochford E.T. (2013). Infection resistance of degradable versus non-degradable biomaterials: An assessment of the potential mechanisms. Biomaterials.

[17] Mayanagi G., Igarashi K., Washio J., Takahashi N. (2017). PH Response and Tooth Surface Solubility at the Tooth/Bacteria Interface. Caries Res..

[18] Spencer P., Ye Q., Misra A., Goncalves S.E., Laurence J.S. (2014). Proteins, pathogens, and failure at the composite-tooth interface. J Dent Res..

[19] Grumezescu A.M., Chifiriuc C.M. (2014). Prevention of microbial biofilms—The contribution of micro and nanostructured materials. Curr. Med. Chem..

[20] Gu J.D. (2003). Microbiological deterioration and degradation of synthetic polymeric materials: recent research advances. Int. Biodeter. Biodegr..

[21] Khvostenko D., Salehi S., Naleway S.E., Hilton T.J., Ferracane J.L., Mitchell J.C., Kruzic J.J. (2015). Cyclic mechanical loading promotes bacterial penetration along composite restoration marginal gaps. Dent. Mater..

[22] Loguercio A.D., Reis A., Bortoli G., Patzlaft R., Kenshima S., Kenshima S. (2006). Influence of adhesive systems on interfacial dentin gap formation in vitro. Oper. Dent..

[23] Awliya W.Y., El-Sahn A.M. (2008). Leakage pathway of Class V cavities restored with different flowable resin composite restorations. Oper. Dent..

[24] Fucio S.B.P., Carvalho F.G., Sobrinho L.C., Sinhoreti M.A.C., Puppin-Rontani R.M. (2008). The influence of 30-day-old Streptococcus mutans biofilm on the surface of esthetic restorative materials-an in vitro study. J. Dent..

[25] Leprince J.G., Palin W.M., Hadis M.A., Devaux J., Leloup G. (2012). Progress in dimethacrylate-based dental composite technology and curing efficiency. Dent. Mater..

[26] Gonçalves L., Filho J.D., Guimarães J.G., Poskus L.T., Silva E.M. (2008). Solubility, salivary sorption and degree of conversion of dimethacrylate-based polymeric matrixes. J. Biomed. Mater. Res. B App. Biomater..

[27] Khalichi P., Cvitkovitch D.G., Santerre J.P. (2004). Effect of composite resin biodegradation products on oral streptococcal growth. Biomaterials..

[28] Cheng L., Zhang K., Weir M.D., Melo M.A., Zhou X., Xu H.H. (2015). Nanotechnology strategies for antibacterial and remineralizing composites and adhesives to tackle dental caries. Nanomedicine.

[29] Melo M.A.S., Guedes S.F.F., Xu H.H.K., Rodrigues L.K.A. (2013). Nanotechnology-based restorative materials for dental caries management. Trends Biotechnol..

[30] Zhang J.F., Wu R., Fan Y., Liao S., Wang Y., Wen Z.T., Xu X. (2014). Antibacterial dental composites with chlorhexidine and mesoporous silica. J. Dent. Res..

[31] Chatzistavrou X., Fenno J.C., Faulk D., Badylak S., Kasuga T., Boccaccini A.R., Papagerakis P. (2014). Fabrication and characterization of bioactive and antibacterial composites for dental applications. Acta. Biomater..

[32] Fraise A.P., Maillard J.Y., Sattar S. (2013). Types of Antimicrobial Agents. Russell, Hugo and Ayliffe’s Principles and Practice of Disinfection, Preservation and Sterilization.

[33] Teughels W., Van Assche N., Sliepen I., Quirynen M. (2006). Effect of material characteristics and/or surface topography on biofilm development. Clin. Oral Implant. Res..

[34] Hori K., Matsumoto S. (2010). Bacterial adhesion: From mechanism to control. Biochem. Eng. J..

[35] Zhang K., Melo M.A.S., Cheng L., Weir M.D., Bai Y., Xu H.H. (2012). Effect of quaternary ammonium and silver nanoparticle-containing adhesives on dentin bond strength and dental plaque microcosm biofilms. Dent. Mater..

[36] Cheng L., Zhang K., Melo M.A.S., Weir M.D., Zhou X., Xu H.H. (2012). Anti-biofilm Dentin Primer with Quaternary Ammonium and Silver Nanoparticles. J. Dent. Res..

[37] Rai M., Yadav A., Gade A. (2009). Silver nanoparticles as a new generation of antimicrobials. Biotechnol. Adv..

[38] Beyth N., Yudovin-Farber I., Bahir R., Domb A.J., Weiss E.I. (2006). Antibacterial activity of dental composites containing quaternary ammonium polyethylenimine nanoparticles against Streptococcus mutans. Biomaterials.

[39] Imazato S. (2003). Antibacterial properties of resin composites and dentin bonding systems. Dent. Mater..

[40] Xu X., Wang Y., Liao S., Wen Z.T., Fan Y. (2012). Synthesis and characterization of antibacterial dental monomers and composites. J. Biomed. Mater. Res. Part B Appl. Biomater..

[41] Cheng L., Weir M.D., Xu H.H., Antonucci J.M., Kraigsley A.M., Lin N.J., Lin-Gibson S., Zhou X.D. (2012). Antibacterial amorphous calcium phosphate nanocomposites with a quaternary ammonium dimethacrylate and silver nanoparticles. Dent. Mater..

[42] Li F., Weir M.D., Xu H.H. (2013). Effects of quaternary ammonium chain length on antibacterial bonding agents. J. Dent. Res..

[43] Simoncic B., Tomcis B. (2010). Structures of novel antimicrobial agents for textiles—A review. Textile. Res. J..

[44] Zhang K., Cheng L., Weir M.D., Bai Y.X., Xu H.H. (2016). Effects of quaternary ammonium chain length on the antibacterial and remineralizing effects of a calcium phosphate nanocomposite. Int. J. Oral. Sci..

[45] Yu P., Wang C., Zhou J., Jiang L., Xue J., Li W. (2016). Influence of Surface Properties on Adhesion Forces and Attachment of Streptococcus mutans to Zirconia In Vitro. Biomed Res Int..

[46] Ishihara K., Ueda T., Nakabayashi N. (1990). Preparation of Phospholipid Polylners and Their Properties as Polymer Hydrogel Membranes. Polym. J..

[47] Zhao X., Zhang Z., Pan F., Ma Y., Armes S.P., Lewis A.L., Lu J.R. (2005). Solution pH-regulated interfacial adsorption of diblock phosphorylcholine copolymers. Langmuir.

[48] Cheng G., Zhang Z., Chen S.F., Bryers J.D., Jiang S.Y. (2007). Inhibition of bacterial adhesion and biofilm formation on zwitterionic surfaces. Biomaterials.

[49] Mashaghi S., Jadidi T., Koenderink G., Mashaghi A. (2013). Lipid Nanotechnology. Int. J. Mol. Sci..

[50] Lewis A.L., Tolhurst L.A., Stratford P.W. (2002). Analysis of a phosphorylcholine-based polymer coating on a coronary stent pre- and post-implantation. Biomaterials..

[51] Takahashi N., Iwasa F., Inoue Y., Morisaki H., Ishihara K., Baba K. (2014). Evaluation of the durability and antiadhesive action of 2-methacryloyloxyethyl phosphorylcholine grafting on an acrylic resin denture base material. J. Prosthet. Dent..

[52] Zhang N., Chen C., Melo M.A., Bai Y., Cheng L., Xu H.H. (2015). A novel protein-repellent dental composite containing 2-methacryloyloxyethyl phosphorylcholine. Int. J. Oral. Sci..

[53] Li F., Weir M.D., Fouad A.F., Xu H.H. (2014). Effect of salivary pellicle on antibacterial activity of novel antibacterial dental adhesives using a dental plaque microcosm biofilm model. Dent. Mater..

[54] Zhang N., Weir M.D., Romberg E., Bai Y., Xu H.H. (2015). Development of novel dental adhesive with double benefits of protein-repellent and antibacterial capabilities. Dent. Mater..

[55] Zhang N., Ma J., Melo M.A., Weir M.D., Bai Y., Xu H.H. (2015). Protein-repellent and antibacterial dental composite to inhibit biofilms and caries. J. Dent..

[56] Mandracci P., Mussano F., Ceruti P., Pirri C.F., Carossa S. (2015). Reduction of bacterial adhesion on dental composite resins by silicon-oxygen thin film coatings. Biomed. Mater..

[57] Goldman G., Starosvetsky J., Armon R. (2009). Inhibition of biofilm formation on UF membrane by use of specific bacteriophages. J. Membrane. Sci..

[58] Rodrigues L., Van Der Mei H., Banat I.M., Teixeira J., Oliveira R. (2006). Inhibition of microbial adhesion to silicone rubber treated with biosurfactant from Streptococcus thermophilus A. FEMS Immunol. Med. Microbiol..

[59] Thorat S.B., Diaspro A., Salerno M. (2014). In vitro investigation of coupling-agent-free dental restorative composite based on nano-porous alumina fillers. J. Dent..

[60] Thorat S.B., Diaspro A., Scarpellini A., Povia M., Salerno M. (2013). Comparative study of loading of anodic porous alumina with silver nanoparticles using different methods. Materials.

